# Imagining the impossible before breakfast: the relation between creativity, dissociation, and sleep

**DOI:** 10.3389/fpsyg.2015.00324

**Published:** 2015-03-26

**Authors:** Dalena van Heugten - van der Kloet, Jan Cosgrave, Harald Merckelbach, Ross Haines, Stuart Golodetz, Steven Jay Lynn

**Affiliations:** ^1^Sleep and Circadian Neuroscience Institute, Nuffield Department of Clinical Neurosciences, University of OxfordOxford, UK; ^2^Forensic Psychology Section, Department of Clinical and Psychological Science, Maastricht UniversityMaastricht, Netherlands; ^3^Department of Statistics, University of OxfordOxford, UK; ^4^Oxford Smart Specs Group, Nuffield Department of Clinical Neurosciences, University of OxfordOxford, UK; ^5^Laboratory of Consciousness and Cognition, Department of Psychology, Binghamton University, State University of New YorkBinghamton, NY, USA

**Keywords:** dissociation, creativity, dreaming, photography, hyperassociativity

## Abstract

Dissociative symptoms have been related to higher rapid eye movement sleep density, a sleep phase during which hyperassociativity may occur. This may enhance artistic creativity during the day. To test this hypothesis, we conducted a creative photo contest to explore the relation between dissociation, sleep, and creativity. During the contest, participants (*N* = 72) took one photo per day for five consecutive days, based on specific daily themes (consisting of single words) and the instruction to take as creative a photo as possible each day. Furthermore, they completed daily measures of state dissociation and a short sleep diary. The photos and their captions were ranked by two professional photographers and two clinical psychologists based on creativity, originality, bizarreness, and quality. We expected that dissociative people would rank higher in the contest compared with low-dissociative participants, and that the most original photos would be taken on days when the participants scored highest on acute dissociation. We found that acute dissociation predicted a higher ranking on creativity. Poorer sleep quality and fewer hours of sleep predicted more bizarreness in the photos and captions. None of the trait measures could predict creativity. In sum, acute dissociation related to enhanced creativity. These findings contribute to our understanding of dissociative symptomatology.

## Introduction

Dissociative symptoms are notorious for their enigmatic nature and include phenomena that encompass excessive daydreaming, memory problems, severe absentmindedness, and impairments, and discontinuities in perceptions of the self, identity, and the environment (Bernstein and Putnam, 1986). Mild dissociative symptoms, related to absorption, and occasional experiences of depersonalization, are not uncommon in the general population, but relatively rare disorders such as dissociative identity disorder and dissociative amnesia represent severe manifestations of psychopathology (Lynn et al., 2012).

Recent studies have linked dissociative symptoms to vivid dreaming, nightmares, and other unusual sleep experiences (Van der Kloet et al., 2012; Van Heugten–Van der Kloet et al., 2013, 2014). However, the famous 19th century British neurologist Hughlings Jackson was the first to view dissociation as the uncoupling of normal consciousness, which results in what he termed ’the dreamy state’ (Meares, 1999). Interestingly, a century later, Levitan (1967, p. 157) hypothesized that “depersonalization is a compromise state between dreaming and waking.”

Previous research has addressed the connection between dissociative symptoms and sleep disturbances. For example, in a recent study (Van Der Kloet et al., 2013), we assayed dissociative symptoms and EEG sleep parameters and found that lengthening of rapid eye movement (REM) sleep predicts dissociative symptoms. Germane to this finding is the idea that the progression of waking state to REM sleep is marked by an increase in “fluid” and hyperassociative thinking (Stickgold et al., 2001), which is a type of mentation also observed in certain dissociative individuals (Van Heugten–Van der Kloet et al., 2013; Lynn et al., 2014).

**FIGURE 1:**
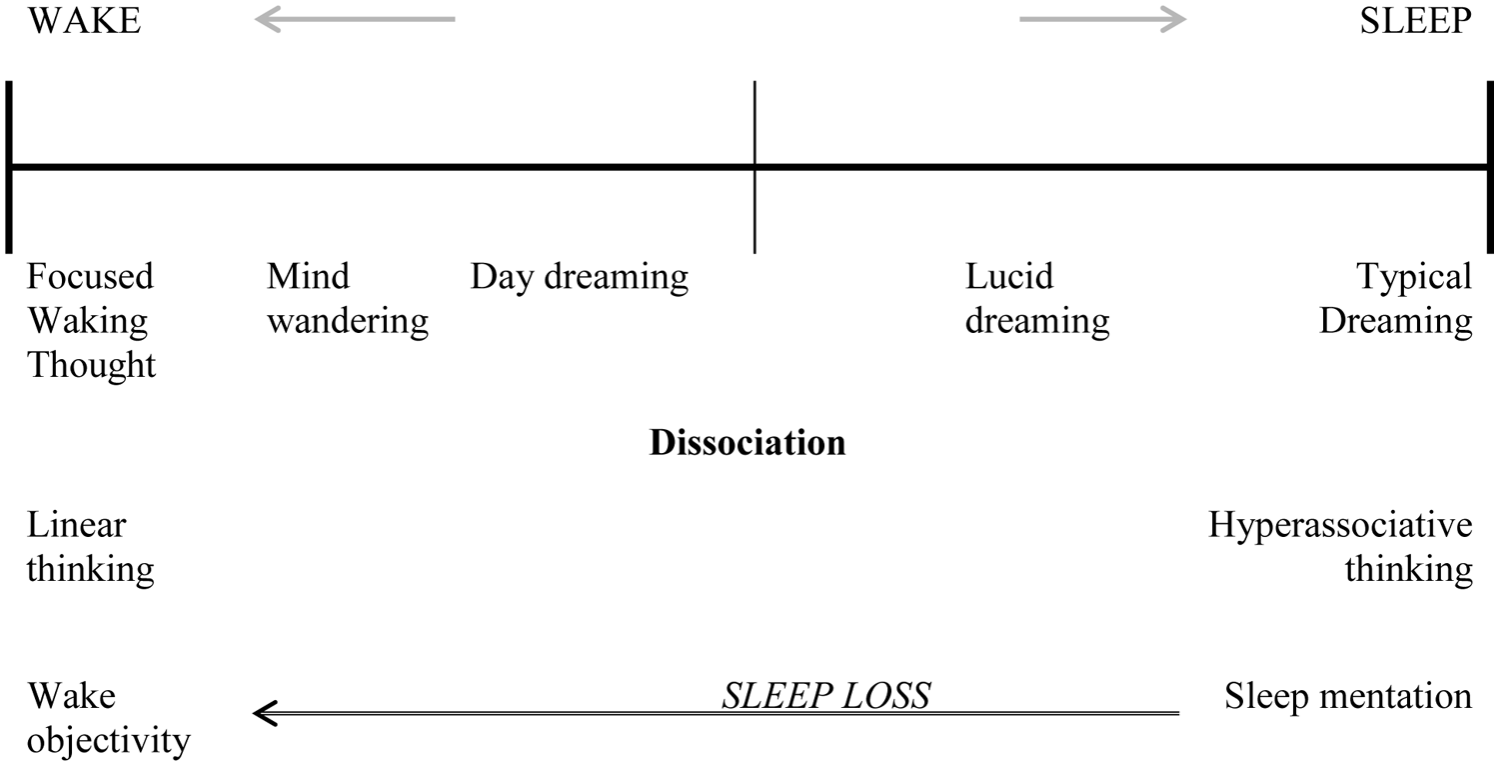
**Consciousness across the sleep-wake cycle depicted as a continuum.** Sleep loss or sleep disturbances may ‘push’ sleep mentation more toward the waking end of the continuum, fostering less linear thinking and the perception of a loss of self during wake, setting the stage for dissociative symptoms.

Rapid eye movement sleep is marked by an increase in cholinergic transmission, similar to wakefulness. At the same time, noradrenergic firing is inhibited during REM sleep, whereas all modulatory neurons slow down but keep firing during non-REM sleep. Accordingly, REM sleep appears to be an ideal state for hyperassociative connections to be made during dreaming. Waking consciousness involves many types of cognitions, such as daydreaming and mind wandering, which in excess could be considered dissociative in nature, (e.g., Foulkes and Fleisher, 1975). Domhoff (2011) describes these cognitions as the ‘default network’ of waking cognition (i.e., mental activity when we are not focusing our thoughts). Hartmann (2010) and Montangero (2012) have argued that thought across the sleep–wake cycle should be regarded as a continuum, with typical dreaming on the one end, focused waking thought on the other end, and daydreaming and mind wandering in between (see **Figure 1**). Indeed, Solms (1999) argued that “Mental state is a constantly negotiated compromise between the poles of waking (…) and dreaming.”

Waking and dreaming consciousness could be viewed as a continuum with experiences such as dissociation and lucid dreaming conceptualized in the middle range. Kahan and LaBerge (1996, 2011) and Kahan et al. (1997) have shown that while there is a reduction in volition in dreaming compared to waking cognition, other properties of thought such as reflective awareness are no more present in waking thought than dreaming. Thus, waking consciousness is capable of the associative thinking thought to be typical of dreaming, and dreaming is capable of the directed and reflexive thinking thought to be typical of waking cognition. We suggest that sleep disturbance could ‘push’ waking consiousness more toward the ‘dreaming’ end of the continuum, fostering less linear, and highly associative thinking during waking (see **Figure 1**). Indeed, loosening of associations and lack of insight are considered to be key elements of schizophrenia (Bleuler et al., 1911/1950; Carroll et al., 1999). This fits well with Hobson’s (2002) view of differentation between waking and dreaming as “a constantly negotiated compromise” (Hobson, 2002, p. 102).

Llewellyn (2011) has advanced an intriguing theory which posits that a progressive de-differentation of wake and dream states of consciousness eventually results in schizophrenia. Because of the differences in neuromodulatory systems between waking and dreaming, the self and the world during dreaming appear to differ from during waking. The perceived world during waking depends on making sense of external sensory input, which engenders a strong sense of external reality in which the ‘self and its inner-world’ exists. In contrast, the world during dreaming makes less distinction between subjectivity and objectivity. A self-organizing system allows for the self and the world in both states to be integrated. Accordingly, when a degree of loss of this self-organization occurs, de-differentation of the waking and dreaming states may ensue, fostering a perception of loss of self, as occurs during episodes of depersonalization.

Creativity involves the understanding of novel ideas and relations; originality and flexibility are two key features of creativity. Chakravarty (2010) suggests that for creativity to occur, we need to be able to engage in divergent thinking, novelty seeking behavior, and suppress latent inhibition to some extent. Most importantly, a creative brain is highly interconnected (both inter- and intrahemispherically), with divergent thinking promoting more connections to be forged through development of new synapses. Relatedly, Faust-Socher et al. (2014) found enhanced creative thinking under dopaminergic therapy in patients with Parkinson’s disease. They proposed that dopamine reduced latent inhibition, thereby enhancing creativity by loosening the associative networks (Faust-Socher et al., 2014). Furthermore, Walker et al. (2002) observed that subjects who were awakened from REM sleep displayed more cognitive flexibility (a 32% advantage), as measured by the number of anagram puzzles solved, than those awakened from NREM sleep.

Rapid eye movement sleep may pose as an ideal state to facilitate creative thinking, with mild frontal dysfunction and high interconnectness through associative networks. We suggest that in highly dissociative people who experience more vivid dreams and other unusual sleep experiences (Van der Kloet et al., 2012), associative and less linear thinking could occur more frequently during waking consciousness, facilitating the emergence of creativity during waking consciousness. Moreover, our research indicates that dissociative symptoms are closely related to fantasy proneness (Rauschenberg and Lynn, 1995; Merckelbach et al., 1999; Van der Kloet et al., 2012), a generally benign trait characterized by deep, profound, and longstanding involvements in fantasy and imagery. The connection between vivid dreaming, fantasy proneness, and dissociation implies that creativity might be a personality trait strongly related to these constructs. Nevertheless, scientific reports on the association between creativity and dissociation are scarce (but see Perez-Fabello and Campos, 2011; Thomson and Jaque, 2012). Accordingly, an important goal of the present study is to examine the relation of creativity and dissociation.

One difficulty in testing the conceptual overlap between dissociative symptoms, fantasy proneness, and creativity is the potential bias introduced by the tendency to overendorse atypical answer options. That is, these constructs might correlate not because they are inherently related but because their overlap is the spurious result of overendorsement (Merckelbach et al., 2014). To circumvent this problem, in the present study we assessed creativity independent of self-reports. More specifically, we conducted a creative photo contest and asked participants to take 5 photos on 5 days that conformed to a designated theme. We chose the photo task rather than a divergent thinking task (e.g., anagram solving), as the former task would be more congruent with the visual nature of dreaming. We invited professional photographers and two clinical psychologists to rate the photos on creativity, originality, bizarreness, and quality. We hypothesized that: (1) highly dissociative people will rank higher in the contest than low-dissociative participants; (2) the most original photos will be taken on days in which the participant scored highest in acute dissociation; (3) unusual content of the photo or description will be more closely related to higher than lower dissociation levels; and (4) lower sleep quality (SQ) and more dream recall will be related to higher rankings on dissociation.

## Materials and Methods

### Participants and Procedure

Ninety two participants (55 women) were recruited via local email and poster advertisements. Inclusion criteria entailed an age of 18 years and older, proficiency in the English language, and access to either a camera or a mobile phone with camera function.

First, participants completed a baseline screening (see Measures) and were asked to compete in a photo contest. During the contest, they took 1 photo each day with a specific theme, for 5 consecutive days. Each morning, they received an e-mail with the assignment for that day. The assignments of the 5 days were always one word (day1: three; day 2: green; day 3: freedom; day 4: desire; day 5: home). Participants uploaded their photo via a password-protected website with a short description to accompany it. The assignment was to take a photo as creative as possible, adhering to the theme. The quality of the photo or the camera, or their professional technique was not important for the competition. Participants also completed the *Clinician-Administered Dissociative States Scale* (CADSS) and a short *sleep diary* every day in the morning (see Measures).

Next, the photos were ranked by two professional photographers and two clinical psychologists based on creativity, originality, and professional technique. We created an online photo ranking system that allowed the judges to view all of the photos for a particular day and rank them in a single session. The web site presented a viewing area to show the current photo and controls that permitted the judges to rate the photo or switch to the previous/next photo in the sequence. A thumbnail view was also presented to allow quick jumps to other photos in the sequence for comparison purposes.

The judges rated all photos on a scale between 0 (least creative) and 10 (most creative). They were asked to briefly view all submissions first, and then to rate each photo relative to all the other photos. They rated at least 1 photo with a grade of 0 and 1 photo with a grade of 10, and all other photos were scored with a grade in between, which produced a ranked list. The photos and their captions were also ranked on the basis of unusual content by the two clinical psychologists, who were selected as judges due to their specialties in imagery rehearsal and fantasy proneness. The participants with the most creative photos on most days won a prize (first prize:£250 Amazon voucher; second prize: £50 Amazon voucher) and their photos will be published in the university newsletter and other media sources. Only the creativity ranking of the photos determined the winner of the contest.

This study was conducted according to the code of ethics on human experimentation established by the Declaration of Helsinki (1964) and amended in Seoul (2008). Approval for the study was obtained from the Medical Sciences Inter Divisional Research Ethics Committee of the University of Oxford (MSD-IDREC-C1-2014-068).

### Measures

*Dissociative Experiences Scale* *II* (DES; Cronbach’s α = 0.94; all α’s from current study). The DES-II (Bernstein and Putnam, 1986; Bernstein-Carlson and Putnam, 1993) is a self-report scale of trait dissociation. Participants indicate on 100 mm visual analog scales (anchors: 0 = never; 100 = always) to what extent they experience 28 dissociative experiences in daily life. Van IJzendoorn and Schuengel (1996) provide meta-analytic evidence for the sound psychometric properties of the DES.

*Iowa Sleep Experiences Survey (ISES*; Cronbach’s α = 0.87). The 18-item ISES (Watson, 2001) assesses the frequency of various sleep- and dream-related experiences, which are rated on a 7-point scale (anchors: 1 = never, 7 = several times a week). The ISES consists of two subscales that measure general sleep experiences and lucid dreaming. It possesses acceptable internal consistency (coefficient α = 0.85; Watson, 2001).

*Gough Creative Personality Scale* (CPS; Cronbach’s α = 0.67). The CPS (Gough, 1979) is a self-report measure of creativity. It consists of 30 adjectives (e.g., capable, humorous, inventive) and the participant is asked to tick all the self-descriptive adjectives. Specific items reflect higher creativity, with higher total scores indicating greater creativity. Kaduson and Schaefer (1991) have reported on the high concurrent validity of the CPS.

*Creative Experiences Questionnaire (CEQ;* Cronbach’s α = 0.82; Merckelbach et al., 2001). The 25-item CEQ measures fantasy proneness. The yes/no items tap daydreaming, intense fantasies, and imagination. The items were derived from case vignettes on fantasy proneness provided by Wilson and Barber (1983). Illustrative items are “I spent more than half of the day on fantasizing or daydreaming” and “Many of my fantasies are as vivid as a good movie.” A total CEQ score is obtained by summing the items endorsed. Merckelbach et al. (2001) found adequate test–retest reliability and internal consistency.

*Clinician-Administered Dissociative States Scale* (Cronbach’s α = 0.91-0.94). The 27-item CADSS (Bremner et al., 1998) is composed of 19 subject-rated items and eight oberver-scored items. Items are scored on a 5-point scale (0 = not at all, 4 = extremely). Bremner et al. (1998) found the CADSS to be a highly reliable and valid instrument to measure present-state dissociative symptoms. In the current study, we administered only the self-report items.

The *sleep diary* is a self-report instrument ^1^ that examines sleep patterns, adapted from the Consensus Sleep Diary (Carney et al., 2012), and contains questions about sleep patterns (e.g., time of lights out, number of awakenings during the night, time of final awakening, and time out of bed), and dream occurrence. We computed sleep pattern variables as follows: SQ was defined as the proportion of time asleep during the sleep period, calculated as the total sleep time divided by the time spent in bed, multiplied by 100. We also computed the average hours of sleep per night across the 5 days (HS). Participants completed the items every morning. Sleep diaries are a commonly used and valid means of collecting data regarding daily activities and sleep perceptions (Cheek et al., 2004).

### Data Analysis

Statistical analyses were performed within the R statistical environment (R Core Team, 2013). Cronbach’s α values were calculated to estimate internal consistency of the baseline and state measures. Pearson product-moment correlations between baseline and state measures were calculated. State and trait data were analyzed using mixed-effects models and regression analyses.

## Results

### Descriptives

Of the 92 participants, 72 participants (55 women; 15 men; 2 unreported) completed all assessments on all days and submitted all 5 photos. Their mean age was 35.8 years (SD = 16.9) and mean level of experience with photography was rated as 4.8 (SD = 1.8) out of a maximum score of 8, indicating that participants were interested in photography and regularly took photographs. A majority reported owning a digital bridge or single lens reflex camera, having some understanding of basic camera controls (shutter speed, aperture, ISO etc.), and using these controls to photograph interesting objects and experiences. The majority of participants were thinking about taking up photography as a hobby or interest. During the study, they displayed a mean level of motivation of 3.6 (SD = 1.1) on a 5-point Likert scale ranging from 1 (“Not motivated at all”) to 5 (“Very motivated”). They slept an average of 7.2 h (SD = 1.5) per night during the 5 day study period. **Table 1** displays mean scores and Pearson product-moment correlations of the trait and state variables. We found strong correlations among the trait measures of dissociation, unusual sleep experiences, fantasy proneness, and creative personality.

**Table 1:** Mean scores (SD) and Pearson product-moment correlations of trait and state variables (*N* = 72).

	*Mean* (SD)	*DES*	*ISES*	*CEQ*	*CPS*
*DES*	13.68 (11.48)	–	–	–	–
*ISES*	35.11 (17.00)	0.54^∗^	–	–	–
*CEQ*	9.37 (4.98)	0.73^∗^	0.45^∗^	–	–
*CPS*	12.57 (2.72)	0.31^∗^	0.06	0.41^∗^	–
*CADSS*	0.28 (0.42)	0.63 to 0.75^∗^	0.40 to 0.53^∗^	0.47 to 0.59^∗^	0.21 to 0.35^∗^
*SQ*	2.88 (0.72)	-0.15 to -0.23	-0.31to -0.19^∗^	-0.20 to -0.05	-0.14 to 0.01
*HS*	7.2 (1.5)	-0.16 to 0.07	0.01-0.20	-0.26 to 0.10^∗^	-0.22 to -0.02

DES, Dissociative Experiences Scale; ISES, Iowa Sleep Experiences Survey; CEQ, Creative Experiences Questionnaire; CPS, Gough’s Creative Personality Scale; CADSS, Clinician-Administered Dissociative States Scale; SQ, average sleep quality across the 5 days; HS, average hours of sleep per night across the 5 days. Correlations of state measures displayed in table are ranging from day 1 to day 5. *^∗^p* < 0.05, ^∗^some *p’s* < 0.05.

### Relations Between Creativity and Trait and State Measures

The judgment panel ranked all the photos of the 5 days based on creativity, originality, bizarreness, and quality in adherence to the specific theme of each day. Scores were normally distributed for all raters. Internal reliability analyses as computed with Cronbach’s alpha showed α’s ranging between 0.63 and 0.73, with the exception of α = 0.36 for day 3, displaying moderate consistency between the four raters. These are displayed in **Table 2** alongside Spearman’s rank correlations between the 4 judges’ creativity scores for each participant (averaged across the 5 days). All judge pairings were significantly correlated. Daily Spearman’s rank correlations between the judges’ creativity scores were also high. The judges’ creativity and originality scores (averaged across the 5 days) were highly correlated (*r* = 0.89, *p* < 0.05). These were also high on a day to day basis (*r’s* = 0.51–0.84, all *p’s <* 0.05). Therefore, scores were averaged across raters, and we focused the majority of our analyses on the creativity score.

**Table 2:** Spearman’s rank correlations and internal consistency analyses (measured by Cronbach’s alpha) between the creativity ratings of our 4 judges.

	*Photo1* *r*	*Photo2* *r*	*Clin1* *r*	*Inter-rater reliability across days:* *Cronbach’s α*
*Photo1*				Day 1 = 0.73
*Photo2*	0.46^∗ ^	–	–	Day 2 = 0.63	
*Clin1*	0.60^∗ ^	0.59^∗^	–	Day 3 = 0.36	
*Clin2*	0.48^∗^	0.35^∗^	0.49^∗^	Day 4 = 0.65	
				Day 5 = 0.69	

Photo1, Professional photographer 1; Photo2, Professional photographer 2; Clin1, Clinical psychologist 1; Clin2, Clinical psychologist 2. ^∗^*p* < 0.05

First, we explored the relation between the judges’ creativity rankings and the trait and state measures; see **Table 3** for an overview. Mean state dissociation was significantly correlated with the creativity score (*r* = 0.27, *p* < 0.05), and the originality score (*r* = 0.29, *p* < 0.05), but not with bizarreness (*r* = 0.11, *p* > 0.05) and quality (*r* = 0.19, *p* > 0.05). Second, we examined the data from the daily sleep diary. Less SQ and fewer HS related to more bizareness in the photos of those days (*r* = -0.27, and *r* = -0.29, both *p’s* < 0.05).

**Table 3:** **Correlations between mean state measures and average ratings of judges on creativity, originality, bizarreness, and quality (*N*= 72)**.

	*Crea*	*Orig*	*Bizar*	*Qual*	*CADSS*	*SQ*
*Crea*	–	–	–	–	–	–
*Orig*	0.89^∗^	–	–	–	–	–
*Bizar*	0.53^∗^	0.32^∗^	–	–	–	–
*Qual*	0.79^∗^	0.73^∗^	0.34^∗^	–	–	–
*CADSS*	0.27^∗^	0.29^∗^	0.11	0.19	–	–
*SQ*	-0.14	-0.06	-0.27^∗^	-0.13	-0.21	–
*HS*	-0.08	-0.00	-0.29^∗^	-0.13	0.08	0.24^∗^

Crea, Creativity average rating; Orig, Originality average rating; Bizar, Bizarreness average rating; Qual, Quality average rating; CADSS, Clinician-Administered Dissociative States Scale; SQ, average sleep quality across the 5 days; HS, average hours of sleep per night across the 5 days. ^∗^*p* < 0.05 (Critical value: *r* = 0.23).

Furthermore, participants reported whether they recalled having a dream last night, and whether this dream had a positive or negative emotional salience. As expected, we found a negative correlation between how happy and how bad a dream was (*r* = -0.54, *p* < 0.05). We found no relation between the emotional salience of the dreams and creativity on the following day (*r* = -0.04, and *r* = 0.04 respectively, both *p’s* > 0.05). Similar results were found for orginality (*r* = 0.01, and *r* = 0.03), and bizareness (*r* = -0.03, and *r* = 0.02), all *p’s* > 0.05.

### Finding Predictors of Creativity, Originality, and Bizarreness

In order to further explore the connection between the creativity rankings and the measures collected (see Measures), while allowing for variance both within and between subjects, we fitted mixed-effects models to the daily creativity data. For an overview of mixed-effects modeling, see (for example) Singer and Willet (2009).

First, we considered a random-intercepts model (model 0 in **Table 4**) and a random-intercepts model with a time slope (model 1). The addition of time to the model improved the fit to the data, with Akaike’s information criterion (AIC) decreasing, and the likelihood ratio test also suggesting we retain this predictor.

**Table 4:** Summary of mixed-effects model fits for creativity rankings.

	*Df*		*AIC*	*BIC*	*LogLik*	*Deviance*	*χ^2^*	*Pr (>*χ*^2^)*
*Model 0*	3		1362.1	1373.7	-678.05	1356.1		
*Model 1*	4		1351.4	1366.9	-671.68	1343.4	12.74	<0.001
*Model 2*	6		1353.8	1377.0	-670.88	1341.8	1.62	0.45

		***Estimate (SE)***				***t***	***p***

*Model 3*	5	0.59 (0.26)	1348.4	1367.8	-669.2	1338.4	2.268	< 0.05

Next, we added random slope effects, to give a random intercepts and slopes model (model 2). This allows for the effect of the day of the study on creativity to vary by participant. The AIC increased with this addition, and the likelihood ratio test also suggested to not retain the random slopes.

Finally, we included a covariate for state dissociation (model 3). By comparison to model 1, we found state dissociation worth retaining in the model, supported by the likelihood ratio test as well as a drop in the AIC. We found this model described the data best. It was not incrementally valuable to add HS, or SQ, to the random-intercepts model. Thus, a model with a random effect for each participant (i.e., subject-specific intercepts), and with a population-wide slope for the fixed effect of the day of the contest on their creativity, plus the fixed-effect covariate of state dissociation, was the preferred model^1^.

We repeated these analyses for the originality ranking, which resulted in similar findings. We found that a model with a random effect for each participant, with a population-wide slope for the fixed effect of the day of the contest on their originality, and with a fixed-effect dissociation covariate, was the preferred model to predict originality rankings (*AIC* = 1394.6; *BIC* = 1413.9). However, this was to be expected with the high correlation between creativity and originality in our sample.

Furthermore, we explored whether the trait measures could predict the judges’ rankings, averaged across the 5 days of the contest. We used best-subsets regression to explore potential models for each of our outcome measures. This analysis considers every possible combination of predictors for the outcome rankings. For each outcome, models with the smallest adjusted *R*^2^ are then further considered.

None of the trait measures could predict the average creativity and originality rankings. However, we found that creative personality significantly predicted the bizarreness ranking, *F*(1,68) = 4.05, *p* = 0.05. However, in this model the explained variance in bizarreness was small; *adj R*^2^ = 0.04.

## Discussion

Our study investigated the relations among creativity, dissociation, and unusual sleep experiences. We found evidence for: (a) high correlations between dissociation and unusual sleep experiences and fantasy proneness, consistent with previous research (Knox and Lynn, 2014); (b) a high correlation between fantasy proneness and unusual sleep experiences (see also Van der Kloet et al., 2012; Watson et al., 2015); (c) a moderate correlation between dissociation and creative personality, and (d) no significant correlation between creative personality and unusual sleep experiences. Our findings are in line with Koffel and Watson (2009) who proposed that unusual sleep experiences, dissociation, and schizotypy belong to a common domain. We also found that state but not trait dissociation predicted creativity and originality in that more creative photos were taken on highly dissociative days. The link between trait dissociation and judged photo creativity did not appear to be direct, but rather depended on higher acute dissociative states on the specific days.

Although we found no connection between dissociation and rated photo bizarreness, fewer HS and poorer SQ were related to more bizarreness. These findings concur with the idea that sleep disturbances increase the likelihood of intrusions of sleep mentation into wakefulnes, consistent with previous studies in which sleep deprivation increased dissociative symptoms (Giesbrecht et al., 2007a; Van Heugten–Van der Kloet et al., 2015). Nevertheless, we did not find poorer sleep to be related to higher creativity. For the de-differentiation theory (Llewellyn, 2011) to be supported, we would assume disruptions in consciousness to go both ways. This would lead to disturbances during waking and sleep, thereby fostering loose associations and enhancing creativity. Thus, the link may be more complex and less direct than we anticipated. Perhaps minor sleep fluctuations over a number of days may not be sufficient to produce significant associations between dreaming and creativity, but these links may be more apparent with fluctuations in state dissociation. An alternative explanation for the absence of the link between sleep and creativity could be that bizarreness is a better indicator of associative thinking than artistic creativity, as bizarreness may reflect ‘highly associative leaps’ following sleep disturbance. Nevertheless, this hypothesis should be confirmed by future studies.

We also found that creative personality predicted photo bizarreness, although the explained variance was limited. Although the correlations that we found between state dissociation and creativity ratings and between sleep loss and bizarreness were modest, they, nevertheless, highlight an important point: most previous studies of dissociation, unusual sleep experiences, and fantasy proneness are entirely based on self-reports (e.g., Merckelbach et al., 1999, 2001; Van Heugten–Van der Kloet et al., 2014), which are vulnerable to the criticism that overlap among these constructs may be an artifact of overendorsement of atypical items (e.g., response bias). We sidestepped this problem by using ratings of independent judges and found meaningful correlates of state dissociation and self-reported sleep loss with creativity and bizarreness.

The relations of dissociation to creativity and of sleep loss to bizarreness are harmonious with the idea that hyperassociativity is a key feature of the dissociation-sleep link. That is, sleep loss may ‘push’ sleep mentation toward the waking end of the consciousness continuum, which may, in turn, contribute to both (a) hyperassociative cognition that marks episodes of dissociation and dissociative conditions (Van Heugten–Van der Kloet et al., 2013; see also **Figure 1**) and (b) creative thinking, likewise characterized by fluidity of associations (Chakravarty, 2010).

Several authors have commented on the overlap between dissociative symptoms, psychotic–like experiences (Giesbrecht et al., 2007b), and dreaming (Koffel and Watson, 2009). Interestingly, the associations among the dream self, dissociative symptoms, and abnormal self experiences in schizophrenia is highly complex. The dreaming state is generated as the brain acts as a closed system, detached from the environment (D’Agostino et al., 2013). A disturbance in the feeling of presence of the self situated in the world is considered to be one of the hallmarks of the schizophrenic prodrome. Indeed, it may be an underlying vulnerability marker, as it seems independent of symptom manifestation and is evident in schizotypal clinical conditions as well (Nelson et al., 2009). This sense that the self is alienated from the experience manifests in various forms in schizophrenia and other conditions, most notably as depersonalization and derealization experiences, which are considered core dissociative symptoms found to be correlated with schizotypy (Lynn et al., 2012).

A number of caveats pertinent to the current study merit consideration. Our population was predominantly female, and the sample size was relatively small. We heavily relied on self-report questionnaires and internal consistency was low at one point in the study. Nevertheless, the questionnaires used in this study were all well-validated with good psychometric properties. Although the internal consistency of our raters was moderate-to-high, the photography ratings are, nevertheless, subjective. Finally, the levels of state dissociation in our sample were relatively low and variations in sleep were minor. Accordingly, it may have been difficult for significant changes to emerge due to lack of variability. Future studies could consider a longer study period (e.g., 3 weeks of sleep data would be optimal) and targeting patients with significantly increased dissociation levels in order to increase generalizability.

These caveats aside, our findings contribute to our understanding of dissociative symptomatology. Perhaps the link between dissociation and creativity may be explained as the product of a modicum of de-differentiation between waking and dreaming that may be advantageous insofar as it enhances artistic creativity. Although some people with an attenuated form of psychopathology may be highly talented (e.g., Isaac Newton), psychopathology may arise when waking and sleep/dreaming states become severely de-differentiated. Or, as Kant eloquently wrote: “The lunatic is one who dreams whilst awake” (Freud, 1999).

## Conflict of Interest Statement

The authors declare that the research was conducted in the absence of any commercial or financial relationships that could be construed as a potential conflict of interest.

## Footnotes

1 A copy of this instrument can be obtained from the first author.
